# Surface Sensing for Biofilm Formation in* Pseudomonas aeruginosa*

**DOI:** 10.3389/fmicb.2017.02671

**Published:** 2018-01-09

**Authors:** Chien-Yi Chang

**Affiliations:** School of Chemistry and Biosciences, University of Bradford, Bradford, United Kingdom

**Keywords:** surface sensing, biofilm, *Pseudomonas aeruginosa*, cyclic-di-GMP, type IV pili

## Abstract

Aggregating and forming biofilms on biotic or abiotic surfaces are ubiquitous bacterial behaviors under various conditions. In clinical settings, persistent presence of biofilms increases the risks of healthcare-associated infections and imposes huge healthcare and economic burdens. Bacteria within biofilms are protected from external damage and attacks from the host immune system and can exchange genomic information including antibiotic-resistance genes. Dispersed bacterial cells from attached biofilms on medical devices or host tissues may also serve as the origin of further infections. Understanding how bacteria develop biofilms is pertinent to tackle biofilm-associated infections and transmission. Biofilms have been suggested as a continuum of growth modes for adapting to different environments, initiating from bacterial cells sensing their attachment to a surface and then switching cellular physiological status for mature biofilm development. It is crucial to understand bacterial gene regulatory networks and decision-making processes for biofilm formation upon initial surface attachment. *Pseudomonas aeruginosa* is one of the model microorganisms for studying bacterial population behaviors. Several hypotheses and studies have suggested that extracellular macromolecules and appendages play important roles in bacterial responses to the surface attachment. Here, I review recent studies on potential molecular mechanisms and signal transduction pathways for *P. aeruginosa* surface sensing.

## Introduction

Bacteria attach to surfaces, aggregate and then form organized and sometimes multi-species communities called biofilms. Biofilm development involves a series of events: (i) free-floating planktonic cells; (ii) initial reversible attachment followed by irreversible attachment of sessile cells; (iii) further growth or aggregation of attached bacteria resulting in microcolony formation; (iv) organized structures with a distinct architecture resulting from microcolony development; and (v) dispersal of planktonic bacteria from mature biofilms. When compared to planktonic cells, bacteria within a biofilm with various physiological phenotypes benefit from genetic adaptation, variation, and stochastic gene switching, resulting in greater resistance to environmental challenges, antibiotic infiltration, and the host immune system (Smith, 2005; Stewart and Franklin, 2008; Monds and O’Toole, 2009). Biofilms have been recognized to pose an increasing risk of healthcare-associated infections and microbial dissemination (Percival et al., 2015). Dispersed cells from biofilms are highly virulent and physiologically different from both planktonic and biofilm cells (Chua et al., 2014). A thorough understanding of biofilm biology is imperative to provide better healthcare management. Surface attachment is the first step for biofilm development and requires a bacterial surface apparatus and extracellular macromolecules for physical sensing. I will review recent research advances in the mechanisms and regulatory pathways of surface sensing, specifically in the model microorganism, *Pseudomonas aeruginosa*.

## *P. aeruginosa*: A Model Microorganism for Studies on Biofilms

*P. aeruginosa*, a ubiquitous Gram-negative bacterium, is capable of adapting to versatile environments such as human tissues, environmental surfaces in hospitals, and indwelling medical devices; this situation makes it the leading cause of acute nosocomial pneumonia or sepsis (Mesaros et al., 2007; Winsor et al., 2011; Percival et al., 2015). *P. aeruginosa* is naturally resistant to a wide range of antibiotics, making antibiotic treatment ineffective. Biofilms attached to medical equipment or indwelling catheters may be one of the major reasons that *P. aeruginosa* infections are highly transmissible among hospitalized patients (Hancock and Speert, 2000; Breidenstein et al., 2011; Poole, 2011; Russotto et al., 2015). The International Nosocomial Infection Control Consortium reported that *P. aeruginosa* nosocomial infections have become a worldwide healthcare issue (Rosenthal et al., 2016).

Forming biofilms has been proposed as a strategy for bacteria to survive under unfavorable conditions (Davies, 2003; Olsen, 2015; Moradali et al., 2017). Viable bacteria within biofilms are shielded and supported by the hydrated polymeric matrix known as extracellular polymeric substances (EPSs). The composition and physicochemical properties of EPSs are bacterial-species-dependent and shaped by environments (Flemming and Wingender, 2010). The key components of EPSs in *P. aeruginosa* are exopolysaccharides including Psl, Pel, and alginate, extracellular DNA (eDNA), lipids, and proteins. Extracellular type IV pili (T4P) and flagella also act as structural elements within mature biofilms (van Schaik et al., 2005; Barken et al., 2008). Cup fimbriae play an important role in cell-to-cell interactions in the early stage of biofilm formation (Wei and Ma, 2013). The composition and functions of EPSs in the *P. aeruginosa* biofilm were highlighted in several reviews (Flemming and Wingender, 2010; Moradali et al., 2017).

## The Motile–Sessile Lifestyle Switch

A comparative analysis including three microarray datasets revealed only five upregulated and six downregulated transcripts with more than twofold changes in *P. aeruginosa* in biofilms compared to planktonic cultures (Patell et al., 2010). RNA sequencing technology (RNA-Seq) has revealed a large amount of RNA transcripts with differential expression between bacteria from two different mature developmental biofilms (24 or 48 h of development) compared to different planktonic growth phases (4 or 12 h of culture), including 24 small-regulatory RNAs (sRNAs). That study suggested that gene expression levels at different biofilm maturation stages are dynamic and sRNAs play potential roles in biofilm development. Nonetheless, when compared to other four microarray datasets in different experimental conditions, “only little consistence” has been found across these datasets, suggesting that biofilm-associated transcripts in *P. aeruginosa* are greatly influenced by experimental conditions (Dötsch et al., 2012). A more recent microarray study showed that there are a total of 2504 genes with different expression levels in different spatial regions of mature biofilms (Heacock-Kang et al., 2017). A proteomic study that compared surface-attached *P. aeruginosa* to their unattached counterparts revealed that a total of 616 proteins (258 over-accumulated and 358 under-accumulated proteins) show modified abundance including proteins in two-component systems (TCSs), second messenger systems, outer membrane components, and appendages (Crouzet et al., 2017). These studies suggest that gene expression levels are continuously tuned in bacteria grown in dynamic biofilms.

Cyclic-3′5′-diguanylic acid (c-di-GMP), a universal second messenger in bacteria, is considered a crucial signal for the motility–sessility switch in *P. aeruginosa* (Hengge, 2009; Romling et al., 2013; Valentini and Filloux, 2016). The major determinant of this substantial phenotypic change is the cellular level of c-di-GMP, which increases threefold to fivefold in *P. aeruginosa* grown on an agar surface compared with the liquid culture. Elevated levels of c-di-GMP promote biofilm formation but to inhibit bacterial motility (Kuchma et al., 2012). Two groups of proteins respond to the cellular levels of c-di-GMP. The first group is diguanylate cyclases (DGCs) containing the GGDEF domain for c-di-GMP synthesis from two molecules of GTP. The other is phosphodiesterases (PDEs) containing EAL or HD-GYP domains for c-di-GMP degradation to pGpG or GMP. GGDEF and EAL domains can both exist in the same protein but are activated under different conditions (Valentini and Filloux, 2016).

The *P. aeruginosa* genome has been predicted to encode 18 GGDEF, 5 EAL, 16 GGDEF/EAL, and 3 HD-GYP domain proteins (Galperin, 2005). Several DGCs (SadC, WspR, and YfiN/TpbB) and PDEs (BifA and RocR) are involved in the production of exopolysaccharides and biofilm formation (Kuchma et al., 2007; Merritt et al., 2010; Bernier et al., 2011). The mechanisms via which c-di-GMP mediates regulation of downstream cellular functions include signal–receptor interactions as effectors or transcriptional regulators (Valentini and Filloux, 2016). For example, PelD is a c-di-GMP receptor, and the signal–receptor complex is essential for Pel production in *P. aeruginosa* (Lee et al., 2007). Alg44, a membrane-associated protein, activates alginate polymerization by allosteric binding of c-di-GMP to its PilZ domain (Merighi et al., 2007; Oglesby et al., 2008; Fata Moradali et al., 2015). FleQ, a flagella master transcriptional regulator, represses genes *pel*, *psl*, and *cdr* encoding EPS components and an adhesin at low levels of c-di-GMP but activates the expression of those genes when c-di-GMP binds to FleQ ATP-binding site at high concentrations of c-di-GMP (Hickman and Harwood, 2008; Baraquet et al., 2012; Baraquet and Harwood, 2013; Su et al., 2015). Elevated levels of c-di-GMP also induce the expression of BrlR, a transcriptional activator of two multi-drug efflux pump operons *mexAB*-*oprM* and *mexEF*-*oprN*, resulting in antibiotic resistance in the initial stage of biofilm formation (Gupta et al., 2013, 2014; Liao et al., 2013). A c-di-GMP-binding protein, FimX, may perform a key function in coupling T4P-mediated twitching motility and adhesion to the levels of c-di-GMP by promoting T4P assembly and retraction through binding to PilB ATPase (Jain et al., 2017).

**FIGURE 1:**
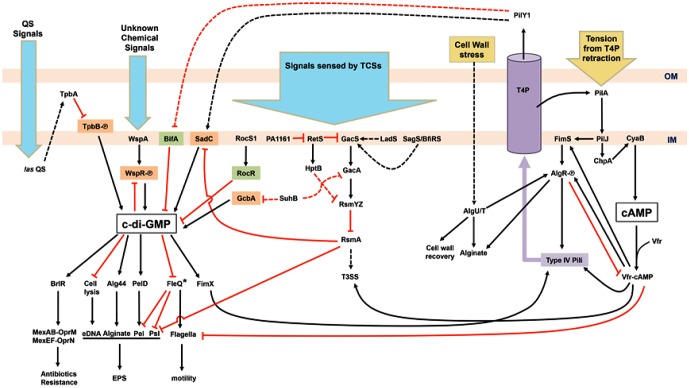
Schematic illustration of the regulatory network of surface sensing in *Pseudomonas aeruginosa*. Upon bacterial attachment to the surface, chemical cues (blue arrows) such as quorum sensing (QS) signals and unknown chemical signals are received by various systems including QS, chemotaxis, and two-component systems (TCSs). Physical contacts (yellow arrows) causing bacterial cell wall stress and retraction of type IV pili (T4P) trigger responding pathways too. Cyclic-di-GMP, the key second messenger for the motile–sessile lifestyle switch, is generated by diguanylate cyclases (orange) and degraded by phosphodiesterases (green). The other second messenger, cAMP, is also involved in the regulation of T4P and flagella biosynthesis. The purple cylinder represents the assembled T4P across the outer membrane (OM) and inner membrane (IM). Phosphorylated proteins are labeled with [scale=0.5]img001. Activation events (black arrowheads) and inhibition events (red T-bars) may be mediated by transcriptional, translational, or post-translational regulatory mechanisms. Dashed arrows or T-bars represent indirect regulation or unknown pathways. Asterisk-labeled FleQ indicates that FleQ plays a dual role as an activator or a repressor in biofilm formation depending on c-di-GMP or ATP binding, respectively.

The changes in levels of c-di-GMP are adjusted in response to extracellular signals received by several sensing systems. Wsp, a chemotaxis-like system consisting of a membrane-bound receptor, WspA, and a regulator, WspR with a GGDEF domain, plays an important role in surface sensing. Signals received by WspA trigger phosphorylation of WspR by the histidine kinase WspE. Phosphorylated WspR synthesizes c-di-GMP and promotes aggregation of WspR to boost the activity of this DGC and the levels of c-di-GMP (Hickman and Tifrea, 2005; Güvener and Harwood, 2007; Huangyutitham et al., 2013). WspR activity can be limited by c-di-GMP binding to an inhibitory site of WspR as negative feedback (De et al., 2008). The connection between c-di-GMP signaling and the Gac–Rsm cascade has been well characterized (**Figure 1**). Free RsmA, a translational repressor protein, promotes a planktonic and more virulent lifestyle through the inhibition of SadC and Psl but upregulation of the Type III secretion system (T3SS) and virulence factors (Irie et al., 2010; Moscoso et al., 2014; Vakulskas et al., 2015). After receiving input signals, the GacSA TCS promotes the expression of two small regulatory RNAs – RsmY and RsmZ – thereby sequestering RsmA (Lapouge et al., 2008; Mikkelsen et al., 2011). Thus, the titration of RsmA by RsmYZ induces the production of sessile determinants. Recently, solution of the nuclear magnetic resonance (NMR) structure of the detector domain of GacS revealed a putative functional pocket for ligand binding and suggested that ligand-induced conformational changes may occur in GacS (Ali-Ahmad et al., 2017). That study provided new insights into the potential mechanisms of surface sensing via GacSA TCS. Several additional sensors and regulators modulate global gene expression levels via the Gac/Rsm system, e.g., SagS/BfiRS, RetS, LadS, PA1161, Hpt, and SuhB/GcbA (**Figure 1**; Mikkelsen et al., 2011; Moscoso et al., 2011; Kong et al., 2013; Vakulskas et al., 2015; Bhagirath et al., 2016; Chambonnier et al., 2016; Valentini and Filloux, 2016; Li et al., 2017). Roc TCS comprising sensor kinase RocS1 and two response regulators, RocA1 and RocR, has been identified because of its regulation in biofilm formation and virulence including Cup fimbriae and T3SS (Kulasekara et al., 2005; Rao et al., 2008). Quorum sensing (QS) is a bacterial intercellular communication mechanism that acts through the production and detection of diffusible signal molecules. In *P. aeruginosa*, two *N*-acyl-homoserine lactone (AHL)-based QS systems, *las* and *rhl*, and one alkylquinolone (AQ)-mediated QS have been identified (Nadal Jimenez et al., 2012). The *las* system upregulates the periplasmic TpbA tyrosine phosphatase, which dephosphorylates YfiN/TpbB, a DGC. Repression of *pel* genes is the result of a lack of c-di-GMP owing to dephosphorylation of TpbB (Ueda and Wood, 2009). Of note, TpbA-dependent c-di-GMP reduction also promotes eDNA release from cell lysis in facilitating biofilm development (Ueda and Wood, 2010). An RNA-Seq study showed that low levels of c-di-GMP induce QS systems and QS-regulated virulence factors via PqsR, the transcriptional regulator of AQ-QS (Lin Chua et al., 2017). Together with c-di-GMP, QS coordinates individual cells at population levels of behavior in response to bacterial population density.

## Extracellular Appendages

The transition from motile to attached cells requires the arrest of flagella-based motility and production of macromolecules for surface attachment. Extracellular appendages act as surface sensors and mediate the first contact with surfaces. Flagella, the major apparatus on the bacterial surface, are not only needed for bacterial motility but also involved in several biological functions. When planktonic bacteria are close to the surface or the viscosity increases, the signal transduction for attachment is triggered as a result of decreased rotation of flagella. This model has been proposed in an earlier study suggesting that the polar flagellum in *Vibrio parahaemolyticus* is the dynamometer of swarming motility and lateral flagella (McCarter et al., 1988). This model in *P. aeruginosa* is partly supported by the inverse regulation between the flagella-mediated swarming and biofilm formation. Flagellar reversal rates are modulated by the chemotaxis cluster IV (CheIV cluster) in response to SadC-BifA adjusted levels of c-di-GMP (Caiazza et al., 2007; Merritt et al., 2007). Nevertheless, it is unclear whether SadC responds to cues of attachment or to the feedback from flagella with reduced rotation.

The T4P is another type of extracellular appendages involved in cell–cell aggregation, biofilm formation, and virulence (Leighton et al., 2015). PilY1, an essential non-pilin protein, along with other pilin subunits, forms a helical pilus fiber (Alm et al., 1996). Two calcium-binding sites in PilY have been revealed, which may be required for controlling pili retraction (Orans et al., 2010) and for binding to integrin of host epithelial cells (Heiniger et al., 2010; Johnson et al., 2011). These findings suggest that the binding of PilY1 to integrins of the target cell enables *P. aeruginosa* to sense attachment and therefore pull itself toward the host. Moreover, surface attachment-induced virulence requires both PilY1 and *las*-mediated QS. PilY1 therefore has been proposed as a mechanosensor for *P. aeruginosa* attachment-induced virulence (Siryaporn et al., 2014). T4P biosynthesis including PilY1 and T4P-mediated twitching motility in *P. aeruginosa* is regulated by cyclic adenosine monophosphate (cAMP) and a chemotactic cluster, PilGHIJK-ChpABC, which is similar to a well-studied Che system in *E. coli* flagella regulation (Baker et al., 2005). The Chp system senses environmental cues and activates the major adenylate cyclase of *P. aeruginosa*, CyaB, for cAMP production, leading to activation of pilus genes via the cAMP-binding protein, a virulence factor regulator (Vfr) (Fulcher et al., 2010). Increased piliation and the expression of PilY1 result in further surface contact and elevation of cAMP levels. Conversely, the expression of flagellar structural components is repressed by high concentrations of cAMP (Wolfgang et al., 2003). Elevated cAMP concentration also promotes the expression of type II and type III secretion systems for further attachment-induced virulence (Siryaporn et al., 2014; Leighton et al., 2015). Moreover, cAMP-Vfr can stimulate the transcription of the *fimS-algR* TCS operon encoding the FimS sensor kinase for AlgR phosphorylation, thus causing activation of the pili operon for T4P assembly and alginate biosynthesis (**Figure 1**; Whitchurch and Alm, 1996; Yu et al., 1997; Whitchurch et al., 2002; Kanack et al., 2006; Belete et al., 2008). After surface contacts, the bacterial cell wall or periplasm may be damaged or stressed, triggering stress pathways for cell wall recovery and surface behaviors. AlgR is also activated by a stress-induced sigma factor σ^22^ (AlgU/T). Together, AlgR and σ^22^ activate the transcription of genes responsible for alginate production and inhibit cAMP-Vfr pathways by repressing *vfr* expression (**Figure 1**; Wood et al., 2006; Wood and Ohman, 2009; Jones et al., 2010).

In a *bifA* mutant, excessive biofilm formation and swarming defect phenotypes (resulting from the failure of c-di-GMP degradation) are suppressed by the second mutation in the *pilY1* gene. In the wild type, increased expression of PilY1 with c-di-GMP produced by SadC suppresses swarming motility. Nonetheless, the global pools of c-di-GMP are not altered in the *pilY1* mutant. Therefore, PilY1 is involved in c-di-GMP regulation related to surface behaviors and functions upstream of SadC-BifA by unknown mechanisms (Kuchma et al., 2010). When bacteria grown on agar surfaces were compared to those grown in liquid broth, expression of *pilY1* increased twofold at transcriptional and translational levels (Luo et al., 2015). A further transposon mutagenesis analysis indicated that *pilY1* is not only regulated by Pil-Chp, cAMP-Vfr, and FimS-AlgR systems but also modulated by T4P assembly. PilJ, a methyl-accepting chemotaxis protein (MCP) in the Pil-Chp system, activates CyaB to produce cAMP when cells are grown on an agar surface. PilJ also interacts directly with FimS thus inducing downstream operons for alginate and pili biosynthesis including the *fimUpilVXY1Y2E* operon. Thus, both cAMP-Vfr and FimS-AlgR coordinate T4P biosynthesis through the input signals from Pil-Chp. PilY1, secreted by the assembled T4P to the outer membrane, then activates SadC resulting in increasing c-di-GMP levels, promoting sessility, and downregulating motility (**Figure 1**; Luo et al., 2015). A recent study indicates that FimV, an inner-membrane protein, with the Chp system via cAMP-Vfr regulates T4P and T4P-regulated twitching motility, which involves repeated extension and retraction of pili mediated by assembly and disassembly of a pilin subunit PilA (Buensuceso et al., 2017). The involvement of FimV and its associated pathways in surface sensing are still unclear. Upon surface contact, attachment and retraction may exert tension on T4P. A bacterial two-hybrid system study suggests that PilJ interacts directly with PilA through their periplasmic domains, suggesting that PilJ may respond to the mechanical tension via PilA (Persat et al., 2015). The mechanical force generated by T4P retraction was measured and found to be ∼30 pN in an experiment with laser tweezers, and the retraction of T4P was not systematically dependent on the levels of c-di-GMP. Nevertheless, c-di-GMP-dependent Pel enhances the T4P-mediated attachment (Ribbe et al., 2017). The shear force in flowing fluid sensed by PiY1 and T4P also provides a cue for elevation of c-di-GMP levels and surface attachment (Rodesney et al., 2017). Thus, mechanosensing via T4P upon initial attachment induces the production of c-di-GMP and c-di-GMP-dependent gene expression via complex regulatory networks and thereby determines bacterial lifestyles on surfaces.

Although several models have been proposed, most biofilm studies have been conducted only with a single material (Tuson and Weibel, 2013). To identify novel anti-biofilm materials, Hook et al. (2012, 2013) employed polymer microarray techniques with various polymer surfaces showing different bacterial responses and biofilm formation on different materials. According to their studies, the bacteria–material interaction cannot be predicted by wettability or surface energy of materials (Alexander and Williams, 2017). Although T4P has been suggested as the major mechanosensing apparatus for surface sensing, how bacteria sense different surfaces and determine attachment is still mostly unknown. It is unlikely that the T4P-mediated surface sensing is the only pathway for responding to every material encountered by bacterial cells, but more likely, unknown pathways or extracellular macromolecules are also involved in the response to attachment to different substrata. A proteomic study of *P. aeruginosa* attached to three different abiotic materials revealed that 785 proteins are common for all three materials, 107 for two of them, and 38 were detected specifically on only one surface (Guilbaud et al., 2017). Accordingly, bacterial responses are specific to attached materials. There is much to learn about bacteria–material interactions.

## Conclusion

I have summarized recent studies on surface sensing and regulatory pathways for biofilm formation in *P. aeruginosa* (**Figure 1**). T4P and its components perform crucial functions in the receipt of external signals and communication between c-di-GMP- and cAMP-associated pathways. The major question in the T4P model is how the secreted PilY1 controls downstream SadC-BifA located on the inner membrane. Sensing of different surface materials is another major question. In addition, chemical characterization or functional groups of materials interacting with key bacterial components (e.g., TCSs) will provide a different angle for studies on bacterial surface sensing. Fundamental multi-disciplinary studies will address these questions, e.g., how bacteria know that they are on a surface, how bacteria distinguish between different materials, and how bacteria respond to different surfaces. Investigating *P. aeruginosa* surface sensing on different substrata will advance our knowledge for identification of effective approaches to elimination of complex biofilms in clinical settings.

## Author Contributions

C-YC drafted the manuscript and prepared the figure.

## Conflict of Interest Statement

The author declares that the research was conducted in the absence of any commercial or financial relationships that could be construed as a potential conflict of interest.
